# Supplementary material for: Species Identification and Profiling of Complex Microbial Communities Using Shotgun Illumina Sequencing of 16S rRNA Amplicon Sequences
Source: PLoS One. 2013 Apr 8;8(4):e60811. doi: 10.1371/journal.pone.0060811 (PMC3620293; doi:10.1371/journal.pone.0060811)
Supplement: File S1 — Supporting figures and tables. (DOC) [file pone.0060811.s001.doc]

# Supplementary Information

**Supplementary Figure S1. Log-scaled version of the plot in Figure 2A.**

**
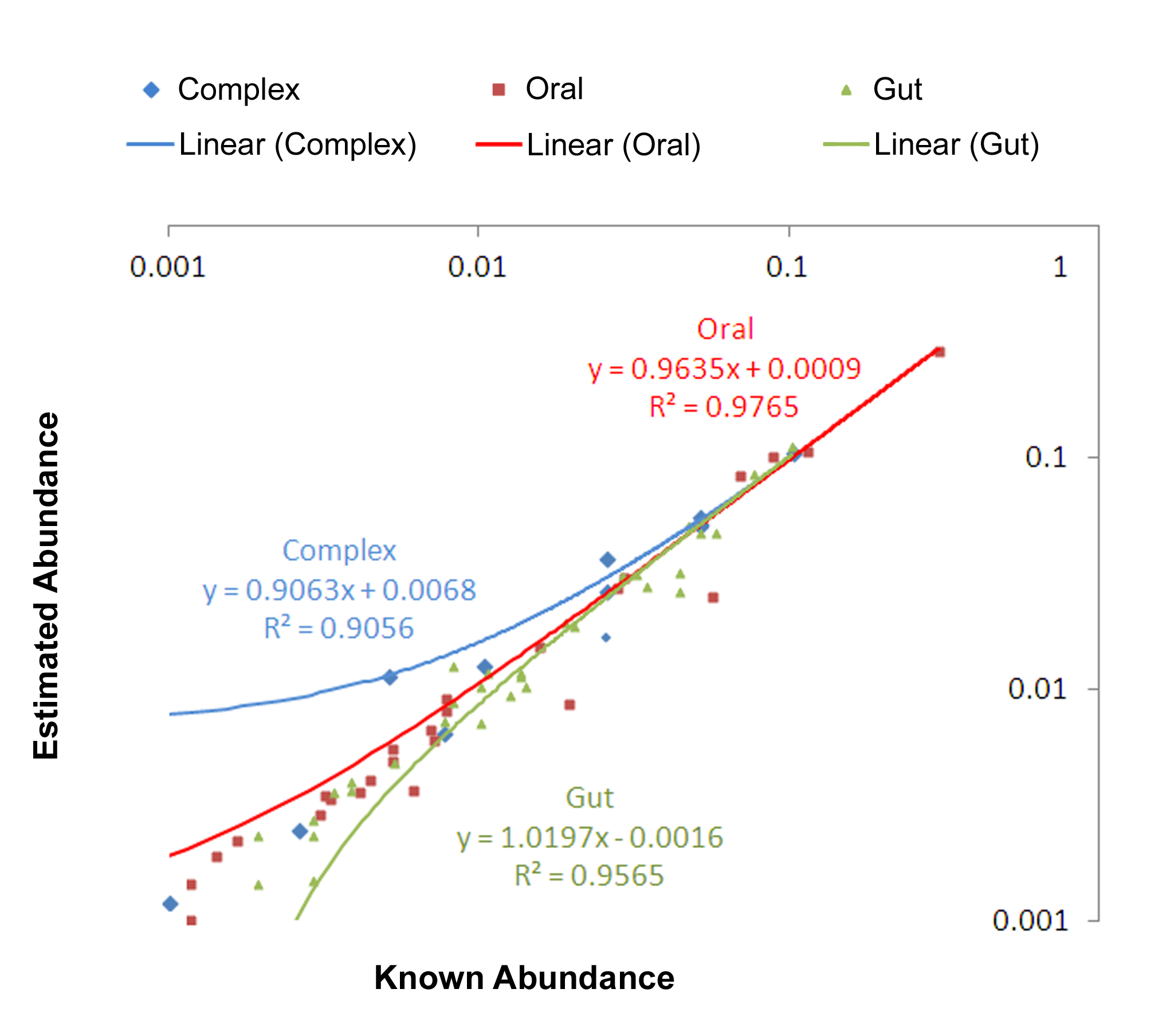
**

**Supplementary Table S1. Constituent bacterial species of ABC33 in alphabetical order.**

*Bacteroides fragilis* JCM 11019T

*Bacteroides thetaiotaomicron* JCM 5827T

*Bacteroides vulgatus* JCM 5826T

*Bifidobacterium adolescentis* JCM 1275T

*Bifidobacterium catenulatum* JCM 1194T

*Bifidobacterium longum* subsp. *infantis* JCM 1222T

*Bifidobacterium longum* subsp. *longum* JCM 1217T

*Blautia coccoides* DSM 935

*Blautia hansenii* JCM 14655T

*Blautia producta* JCM 1471T

*Clostridium difficile* DSM 1296

*Clostridium leptum* DSM 753

*Clostridium perfringens* DSM 756

*Collinsella aerofaciens* JCM 10188T

*Corynebacterium kroppenstedtii* JCM 11950T

*Enterococcus faecalis* ATCC 29212

*Escherichia coli* O157

*Faecalibacterium prausnitzii* DSM 17677

*Lactobacillus acidophilus* JCM 1132T

*Lactobacillus gasseri* JCM 1131T

*Mycobacterium tuberculosis* H37Ra ATCC 25177

*Neisseria gonorrhoeae* ATCC 53423

*Neisseria lactamica* DSM 4691

*Neisseria meningitidis* DSM 10036

*Neisseria mucosa* JCM 12992T

*Neisseria perflava* ATCC 14799

*Nocardia asteroides* JCM 3384T

*Porphyromonas gingivalis* JCM 12257T

*Propionibacterium acnes* JCM 6425T

*Roseburia intestinalis* DSM 14610

*Staphylococcus aureus* ATCC 6538P

*Streptococcus oralis* JCM 12997T

*Streptococcus thermophilus* JCM 20026

**Supplementary Table S2**. **Robustness of EMIRGE results to the number of reads used.** Results shown are for the “Complex” community and at 0.1% relative abundance threshold.

| Number of Reads | Genus-level recall (%) | Genus-level precision (%) | Species-level recall (%) | Species-level precision (%) |
| --- | --- | --- | --- | --- |
| 50K | 59 | 100 | 24 | 82 |
| 100K | 64 | 100 | 32 | 86 |
| 200K | 68 | 100 | 41 | 88 |
| 500K | 64 | 100 | 43 | 89 |

**Supplementary Table S3. Performance of QIIME vs modQIIME. Results shown are for the “Complex” community and at 0.1% relative abundance threshold.**

| Method | Genus-level recall (%) | Genus-level precision (%) | Species-level recall (%) | Species-level precision (%) |
| --- | --- | --- | --- | --- |
| QIIME | 82 | 46 | 46 | 55 |
| modQIIME | 100 | 55 | 59 | 59 |

**Supplementary Table S4.** ***In silico* assessment of PCR primers for the 16S rRNA gene using Greengenes, RDP and SILVA**. Primers with an asterisk appended to their names are optimized versions of the respective canonical primers which have had between 1 to 3 additional positions (underlined for clarity) replaced with degenerate nucleotides to improve their matching rate. The primer numbering is based on the *E. coli* system of nomenclature as in Brosius *et al*. [37].

|  | **Primer** | **Sequence in 5’−3’ orientation** | **Greengenes** | **RDP** | **SILVA** | **Combined** |
| --- | --- | --- | --- | --- | --- | --- |
|  | Size of data set (no. of sequences) | | 1,011,632 | 1,727,996 | 618,442 | 3,358,070 |
| 1. | 8F | AGAGTTTGATCCTGGCTCAG | 125,329 (12%) | 159,533 (9%) | 129,212 (21%) | 414,074 (12%) |
| 2. | 8F* | AGAGTTTGATCMTGGCTCAG | 149,371 (15%) | 201,280 (12%) | 155,132 (25%) | 505,783 (15%) |
| 3. | 338F | ACTCCTACGGGAGGCAGC | 946,219 (94%) | 1,384,005 (80%) | 486,609 (79%) | 2,816,833 (84%) |
| 4. | 338F* | ACTYCTACGGRAGGCWGC | 954,507 (94%) | 1,400,148 (81%) | 494,810 (80%) | 2,849,465 (85%) |
| 5. | 341F | CCTACGGGAGGCAGCAG | 947,239 (94%) | 1,396,163 (81%) | 486,859 (79%) | 2,830,261 (84%) |
| 6. | 533R | GTGCCAGCAGCCGCGGTAA | 910,062 (90%) | 1,342,620 (78%) | 566,000 (92%) | 2,818,682 (84%) |
| 7. | 533R* | GTGCCAGCMGCCGCGGTAA | 918,217 (91%) | 1,347,229 (78%) | 580,549 (94%) | 2,845,995 (85%) |
| 8. | 784F | AGGATTAGATACCCTGGTA | 837,677 (83%) | 1,142,175 (66%) | 435,323 (70%) | 2,415,175 (72%) |
| 9. | 805R | ATTAGATACCCTGGTAGTC | 839,319 (83%) | 1,142,173 (66%) | 436,995 (71%) | 2,418,487 (72%) |
| 10. | 907F | AAACTYAAAKGAATTGACGG | 929,021 (92%) | 1,128,872 (65%) | 535,808 (87%) | 2,593,701 (77%) |
| 11. | 967F | CAACGCGAAGAACCTTACC | 674,109 (67%) | 762,533 (44%) | 319,181 (52%) | 1,755,823 (52%) |
| 12. | 1061R | CRRCACGAGCTGACGAC | 976,162 (96%) | 1,125,505 (65%) | 516,018 (83%) | 2,617,685 (78%) |
| 13. | 1195R | GAGGAAGGYGGGGAYGACGTC | 909,466 (90%) | 1,013,808 (59%) | 459,535 (74%) | 2,382,809 (71%) |
| 14. | 1391F | TGYACACACCGCCCGTC | 339,602 (34%) | 384,798 (22%) | 413,479 (67%) | 1,137,879 (33%) |
| 15. | 1391F* | TGYACWCACYGCCYGTC | 368,947 (36%) | 411,983 (24%) | 444,247 (72%) | 1,225,177 (36%) |
| 16. | 1492F | AAGTCGTAACAAGGTA | 149,137 (15%) | 171,929 (10%) | 162,191 (26%) | 483,257 (14%) |

**Supplementary Table S5**. **Sequencing and analysis protocols in published Illumina-sequencing-based 16S rRNA studies.**

| **Reference** | **V Region** | **Read length** | **Taxonomic Assignment Method** |
| --- | --- | --- | --- |
| Lazarevic *et al*., 2009 | V5 | 76 bp | GAST and the RDP Classifier. |
| Hummelen *et al*., 2010 | V6 | 76 bp | UCLUST clustering and BLAST. |
| Claesson *et al*., 2010 | V1/V2, V2/V3, V3/V4, V4/V5, V5/V6, V7/V8 | 101 bp | RDP Pyrosequencing Pipeline and the RDP naïve Bayesian Classifier. |
| Zhou *et al*., 2010 | V6 | 100 bp | The program *Merger* for merging the PE reads and GAST. |
| Gloor *et al*., 2010 | V6 | 76 bp | UCLUST clustering and BLAST. |
| Caporaso *et al*., 2011 | V4 | 100 bp | QIIME’s wrappers for the RDP classifier. |
| Miller *et al*., 2011 | Genomic | 76 bp | EMIRGE. |
| Bartram *et al*., 2011 | V3 | 125 bp | RDP naïve Bayesian Classifier v.2.1. |
| Degnan & Ochman, 2012 | V6 | 75/100 bp | RDP Pyrosequencing Pipeline. |

**Supplementary Table S6. Diversity metrics for EMIRGE, modQIIME and RTAX on different datasets. The metrics reported here are Chao1 (bias corrected version; [Chao, 1987]) and Shannon Entropy (separated by a comma). Both were computed with QIIME’s alpha_diversity.py script with options '-m chao1' and ‘-m shannon’ respectively. Results from Table 2 were used to calculate the diversity metrics and the known profile was used to compute values for the “True Profile” column.**

| **Dataset** | **True Profile** | **EMIRGE** | **modQIIME** | **RTAX** |
| --- | --- | --- | --- | --- |
| *“Oral”* | 38, 3.9 | 56, 4.2 | 167, 9.3 | 45, 3.3 |
| *“Gut”* | 45, 4.7 | 85, 5.5 | 190, 9.6 | 54, 3.0 |
| *“Complex”* | 29, 4.2 | 28, 4.0 | 189, 8.6 | 58, 3.4 |
| ABC33 | 33, 5.0 | 124, 6.3 | 124, 5.2 | 67, 5.6 |

**Supplementary Table S7. Species identified by EMIRGE analysis of shotgun Illumina sequencing datasets for stool and throat swab samples. Species reported are those with relative abundance greater than 0.005% and the analysis was done with 500K reads (see Supplementary Table S8 for validation at this threshold on *in silico* datasets). Species found in the stool samples which are listed at http://genome.wustl.edu/genomes/list/microorganisms and species found in the throat swabs which are in the Human Oral Microbiome Database (http://www.homd.org) are in bold.**

| Adult Stool | ***Bacteroides uniformis*** (4.4%)*,* ***Eubacterium rectale*** (1.34%)*, Bifidobacterium longum* (1.27%)*,* ***Bacteroides eggerthii*** (0.96)*,* ***Ruminococcus gnavus*** (0.8%)*,* ***Faecalibacterium prausnitzii*** (0.47%)*, Parabacteroides distasonis* (0.42%)*,* ***Bacteroides stercoris*** (0.29%)*,* ***Ruminococcus callidus*** (0.22%)*,* ***Collinsella aerofaciens*** (0.17%)*, Haemophilus parainfluenzae* (0.15%)*,* ***Bifidobacterium adolescentis*** (0.14%)*, Bacteroides massiliensis* (0.12%)*, Coprococcus catus* (0.09%)*, Bacteroides fragilis* (0.05%)*,* ***Actinomyces odontolyticus*** (0.04%)*, Veillonella parvula* (0.03%)*, Clostridium orbiscindens* (0.03%)*, Porphyromonas gingivalis* (0.03%)*,* ***Bacteroides ovatus*** (0.02%)*,* ***Coprococcus eutactus*** (0.02%)*,* ***Holdemania filiformis*** (0.01%)*, Porphyromonas endodontalis* (0.01%)*, Prevotella pallens* (0.01%)*, Eggerthella lenta* (0.01%) |
| --- | --- |
| Infant Stool | ***Ruminococcus gnavus*** (8.3%)*,* ***Clostridium ramosum*** (1.57%)*,* ***Bacteroides ovatus*** (1.54%)*, Clostridium innocuum* (1.47%)*,* ***Bifidobacterium breve*** (1.01%)*, Bacteroides fragilis* (0.93%)*, Blautia producta* (0.71%)*, Parabacteroides distasonis* (0.57%)*,* ***Clostridium spiroforme*** (0.43%)*, Clostridium orbiscindens* (0.28%)*, Streptococcus salivarius* (0.21%)*,* ***Clostridium hylemonae*** (0.18%)*,* ***Anaerostipes caccae*** (0.1%)*, Escherichia fergusonii* (0.09%)*,* ***Bacteroides vulgates*** (0.09%)*, Shigella dysenteriae* (0.09%)*,* ***Actinomyces odontolyticus*** (0.05%)*, Bifidobacterium longum* (0.02%)*, Clostridium sp. MLG480* (0.01%)*, Coprobacillus cateniformis* (0.01%) |
| Throat SW 18 | ***Streptococcus salivarius*** (5.8%)*,* ***Prevotella histicola*** (5.78%)*,* ***Actinomyces odontolyticus*** (4.1%)*,* ***Veillonella dispar*** (3.45%)*,* ***Granulicatella adiacens*** (1.8%)*,* ***Prevotella pallens*** (1.65%)*,* ***Rothia dentocariosa*** (0.72%)*,* ***Gemella sanguinis*** (0.45%)*,* ***Solobacterium moorei*** (0.31%)*,* ***Veillonella parvula*** (0.1%)*,* ***Streptococcus mutans*** (0.06%)*,* ***Lactobacillus salivarius*** (0.05%)*,* ***Corynebacterium matruchotii*** (0.03%)*,* ***Staphylococcus aureus*** (0.03%)*,* ***Bifidobacterium longum*** (0.02%)*,* ***Porphyromonas gingivalis*** (0.01%)*,* ***Scardovia inopinata*** (0.01%)*, Streptococcus thermophilus* (0.01%) |
| Throat 50658 | ***Streptococcus salivarius*** (4.39%)*,* ***Actinomyces odontolyticus*** (3.57%)*,* ***Porphyromonas gingivalis*** (2.84%)*,* ***Veillonella dispar*** (1.9%)*,* ***Prevotella pallens*** (1.31%)*,* ***Porphyromonas endodontalis*** (1.26%)*,* ***Prevotella histicola*** (1.14%)*,* ***Granulicatella adiacens*** (0.85%)*,* ***Tannerella forsythia*** (0.71%)*,* ***Streptococcus anginosus*** (0.46%)*,* ***Solobacterium moorei*** (0.39%)*,* ***Prevotella intermedia*** (0.36%)*,* ***Prevotella tannerae*** (0.29%)*,* ***Treponema denticola*** (0.24%)*,* ***Haemophilus parainfluenzae*** (0.23%)*,* ***Treponema amylovorum*** (0.14%)*,* ***Corynebacterium matruchotii*** (0.13%)*,* ***Abiotrophia defective*** (0.12%)*,* ***Lactobacillus salivarius*** (0.08%)*,* ***Rothia mucilaginosa*** (0.08%)*, Prevotella nanceiensis* (0.08%)*,* ***Veillonella parvula*** (0.06%)*,* ***Selenomonas sputigena*** (0.06%)*,* ***Rothia dentocariosa*** (0.05%)*,* ***Campylobacter rectus*** (0.05%)*,* ***Prevotella nigrescens*** (0.05%)*,* ***Prevotella baroniae*** (0.04%)*,* ***Treponema lecithinolyticum*** (0.03%)*,* ***Corynebacterium durum*** (0.03%)*,* ***Treponema socranskii*** (0.03%)*,* ***Bulleidia extructa*** (0.02%)*,* ***Selenomonas noxia*** (0.01%)*,* ***Streptococcus cristatus*** (0.01%)*, Streptococcus pseudopneumoniae* (0.01%)*, Lactobacillus mucosae* (0.01%) |

**Supplementary Table S8.** **Performance of EMIRGE at a relative abundance threshold of 0.005% using 500K reads.**

| Community | Genus-level recall (%) | Genus-level precision (%) | Species-level recall (%) | Species-level precision (%) |
| --- | --- | --- | --- | --- |
| “Oral” | 80 | 90 | 53 | 94 |
| “Gut” | 86 | 94 | 80 | 100 |
| “Complex” | 71 | 89 | 42 | 92 |
